# Familiarity revealed by involuntary eye movements on the fringe of awareness

**DOI:** 10.1038/s41598-019-39889-6

**Published:** 2019-02-28

**Authors:** Gal Rosenzweig, Yoram S. Bonneh

**Affiliations:** 1 Interdisciplinary graduate authority, University of Haifa, Haifa, Israel; 2 School of Optometry and Vision Science, Faculty of life Sciences, Bar-Ilan University, Ramat-Gan, Israel

## Abstract

Involuntary eye movements during fixation of gaze are typically transiently inhibited following stimulus onset. This oculomotor inhibition (OMI), which includes microsaccades and spontaneous eye blinks, is modulated by stimulus saliency and anticipation, but it is currently unknown whether it is sensitive to familiarity. To investigate this, we measured the OMI while observers passively viewed a slideshow of one familiar and 7 unfamiliar facial images presented briefly at 1 Hz in random order. Since the initial experiments indicated that OMI was occasionally insensitive to familiarity when the facial images were highly visible, and to prevent top-down strategies and potential biases, we limited visibility by backward masking making the faces barely visible or at the fringe of awareness. Under these conditions, we found prolonged inhibition of both microsaccades and eye-blinks, as well as earlier onset of microsaccade inhibition with familiarity. These findings demonstrate, for the first time, the sensitivity of OMI to familiarity. Because this is based on involuntary eye movements and can be measured on the fringe of awareness and in passive viewing, our results provide direct evidence that OMI can be used as a novel physiological measure for studying hidden memories with potential implications for health, legal, and security purposes.

## Introduction

### Involuntary eye movements, oculomotor inhibition, and familiarity

During fixation of gaze, our eyes move involuntarily in a random-walk like movement and occasional small saccades or microsaccades^1,2^. Microsaccade direction and timing have been linked to anticipation, surprise, and attention shifts^3–9^. When consciously perceiving a stimulus onset, even when predictable or illusory^10^, microsaccades are first inhibited for a short period of time, then disinhibited and the rate increases before returning to baseline (see review in^11^). This oculomotor inhibition (OMI) effect, which was found for microsaccades as well as for spontaneous eye blinks^12,13^, depends on the properties of the stimulus, attention, and expectation^11,13–16^; the saliency of the stimulus systematically affects the inhibition time course^13^. Whereas saliency due to sensory bottom-up properties such as visual contrast expedites the inhibition onset and shortens its duration^13,14^, saliency due to perceptual deviance, such as for auditory oddballs, prolongs it^7–9,13,17^. To date, the dependence of OMI on familiarity is not known. Since familiar stimuli tend to stand out and attract attention, they appear salient^18–22^, but it is not yet clear whether this saliency should shorten the inhibition due to the enhanced and possibly faster processing^13,23^, or alternatively prolong it, similarly to the response to oddballs^6,7,14,16^, e.g., due to a richer encoding in working memory^24,25^.

### Faces and familiarity, with and without consciousness

Faces serve as ideal stimuli for investigating the effect of familiarity. Facial stimuli include rich information and their processing is based on the spontaneous activation of a wide range of personal knowledge and emotions^24,26^. Multiple evidence suggest that only familiar faces are processed unconsciously or on the verge of awareness, with evidence from masking paradigms^27,28^, and continuous flash suppression (CFS) in which familiar faces were found to break suppression and reach awareness faster^27^ (see review in^26^). This suggests that a powerful method for dissociating between familiar and unfamiliar faces could be subconscious stimulation or stimulation on the verge of consciousness, creating a threshold or a winner-takes-all effect, with the familiar faces reaching awareness more often, thus exaggerating the difference between the familiar and unfamiliar.

### Face familiarity revealed by eye movements

To date, few studies have investigated the pattern of eye movements when examining familiar and unfamiliar faces presented for only a few seconds. Longer fixation durations were found when the familiar face had to be reported or concealed^25,29,30^. In contrast, shorter fixation durations on familiar faces were recently reported, when observers had to examine and memorize 4 faces for later reporting^31^, presumably because the familiar face requires a shorter memorizing process. These studies, however, do not shed light on OMI because they refer to free viewing of static stimuli and not to the involuntary oculomotor response to visual onsets, which we investigated in the current study. We assumed that OMI would be a better objective measure because it is less prone to top down strategies and potential biases.

### The current study

The aim of the current study was to assess the sensitivity of the oculomotor inhibition (OMI) effect to familiarity, focusing on faces. We expected to find such sensitivity for several reasons. First, familiar compared with non-familiar faces induce a larger P300 ERP response^32^, which is a marker of attention^33–35^; this was also found to be correlated (but not identical) to the OMI for microsaccades^36^. Second, familiar faces stand out among unfamiliar faces^18–22^, and are therefore expected to induce OMI related to deviance. Third, given the evidence for co-variation of OMI and consciousness for simple stimuli at threshold^37^, and the evidence for familiar faces breaking suppression more readily than unfamiliar faces^18–20^, face stimuli on the verge of awareness could create an ideal condition for exaggerating the difference in OMI response between the familiar and unfamiliar, if it exists.

## Results

**Figure 1:**
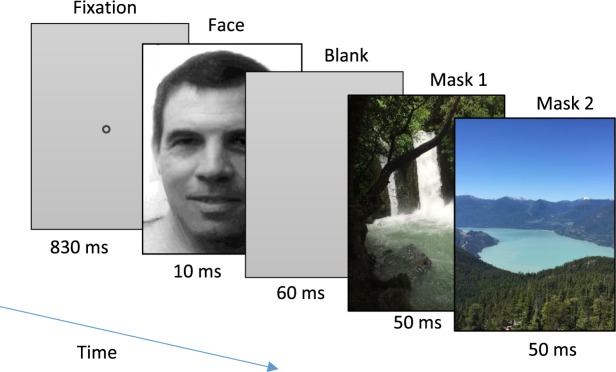
Experimental paradigm, a single trial. In each trial, participants passively viewed a sequence of images as shown, with one face image in gray levels and two mask images in color, presented on a gray background. The temporal sequence is shown from left to right with presentation duration specified for each display. Trials (96 in a run) were repeated automatically at 1 Hz rate. The rapid sequence made the face images barely visible. On each trial a different face was randomly presented – from 1 familiar and 7 non-familiar faces. Participants were asked to pay attention to the stimuli while their eyes were recorded.

In one experiment, observers passively viewed a 1 Hz slide show of briefly flashed facial images, masked by 2 color images that made the faces barely visible (Fig. 1). Eight facial images were presented in random order, one of which was familiar, with a total of 96 presentations per run, 3 runs per participant. Preliminary experiments with six observers using unmasked stimuli indicated that the familiar face often induced longer OMI than the other faces, but this difference was inconsistent, possibly due to the use of top-down strategies or individual biases (Supp. material, Fig. S1). To avoid such effects and increase the consistency, we used masked stimuli for which the observers reported barely noting that the stimuli were faces. The masked stimuli consisted of two kinds of familiar faces: a universally familiar (not mentioned prior to the experiment, 12 participants), and a locally familiar (7 participants not familiar with the first face, see Methods). Since no significant difference was found between the groups, the data were combined. In a set of analyses described next, we compared the involuntary oculomotor response to the familiar face with the response to the unfamiliar faces and quantified the properties and time course of the oculomotor inhibition effect, as described next.

### The effect of familiarity on the microsaccade rate modulation

**Figure 2:**
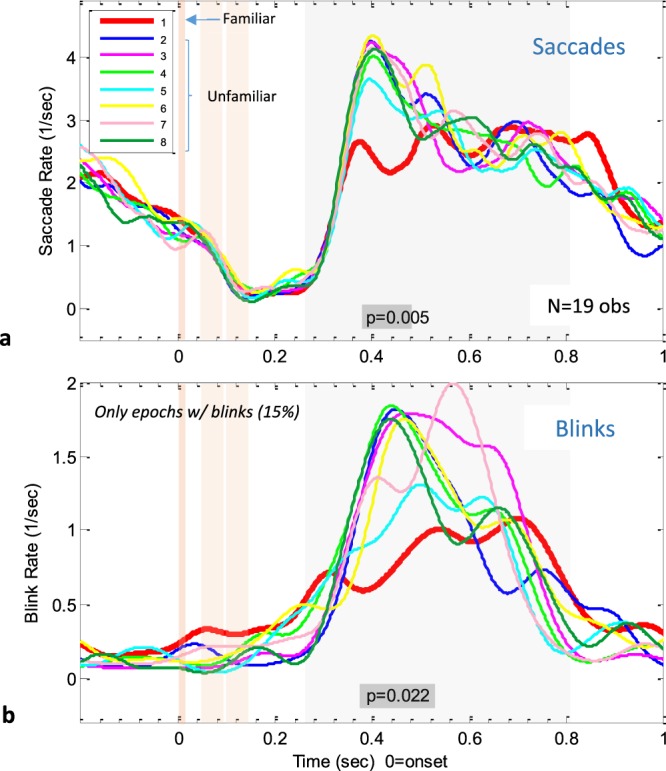
The effect of familiarity on the oculomotor rate modulation. Microsaccade (**a**) and Blink (**b**) rate modulation for the familiar (1, bold red) compared to every non-familiar face (2–8). Data were averaged per face across epochs within observer and then across observers. Time zero represents stimulus onset, with shaded orange areas illustrate the stimulus target and masks times, while the shaded gray area illustrates the time window (250–800 ms) used for subsequent RT analyses. For the Blink rates in (**b**), only epochs with a blink after stimulus onset were included (about 15%). Note the prolonged inhibition for the familiar face (red) for microsaccades as well as blinks. The gray bar indicates the significant cluster showing difference between the familiar face and the average of all other faces (nonparametric permutation test, see Methods), *p = 0.005 for microsaccades, and *p = 0.022 for blinks.

The results for the microsaccade and blink rate modulation are shown in Fig. 2. The group averages across 19 observers are plotted for each of the faces, including the familiar face (#1, in bold), time-locked to the stimulus face image onset. For microsaccades (Fig. 2a), all conditions showed a clear inhibition that started prior to the stimulus onset due to a temporal expectation, followed by a sharp increase in the microsaccade rate around 280 ms post stimulus, reaching a peak around 400 ms, and slowly returning to baseline, roughly similar to the typical pattern of microsaccade inhibition observed in previous studies^13,38^. Critically, the inhibition release in response to the familiar face had a lower peak compared with all unfamiliar faces, deviating from the familiar faces average from around 380 ms until around 500 ms post-stimulus onset. The non-parametric significance test (see Methods) revealed one significant segment of difference around 380–480 ms (p = 0.005). The results for the spontaneous eye blinks (Fig. 2b) showed a similar familiarity effect. We included in the analysis only epochs with one or more post-stimulus blinks, in order to allow averaging across observers and to focus on the main effect, which is only defined when blinks occur. The included epochs were ~15% of the sample, on average, across observers, with a varied percentage between zero (2 observers) and 50%. This implies that the actual blink rate was ~6 times lower than that shown in the figure. It also reduced the number of observers (by 2), but otherwise, the observers were assigned an equal weight in the average regardless of their blinking rate. When comparing microsaccades and blinks, we found that the blinks’ peaks occurred 40 ms later; however, both showed a pattern of deviation from the familiar face rate modulation similar to that of the unfamiliar faces. This deviation appears as a delayed and “smeared” release from inhibition.

We conducted an additional experiment with new observers to verify the visibility level during the experiment and the efficacy of the masking appear (Supp. material, Fig. S2). The results showed that the stimuli were “barely visible” on average. In this experiment we also examined whether the observers perceived and remembered the familiar (but masked) face that was presented to them without any hint for the type of stimuli presented. All the observed reported observing the familiar face.

### The effect of familiarity on microsaccade and blink RT: early and late effects

**Figure 3:**
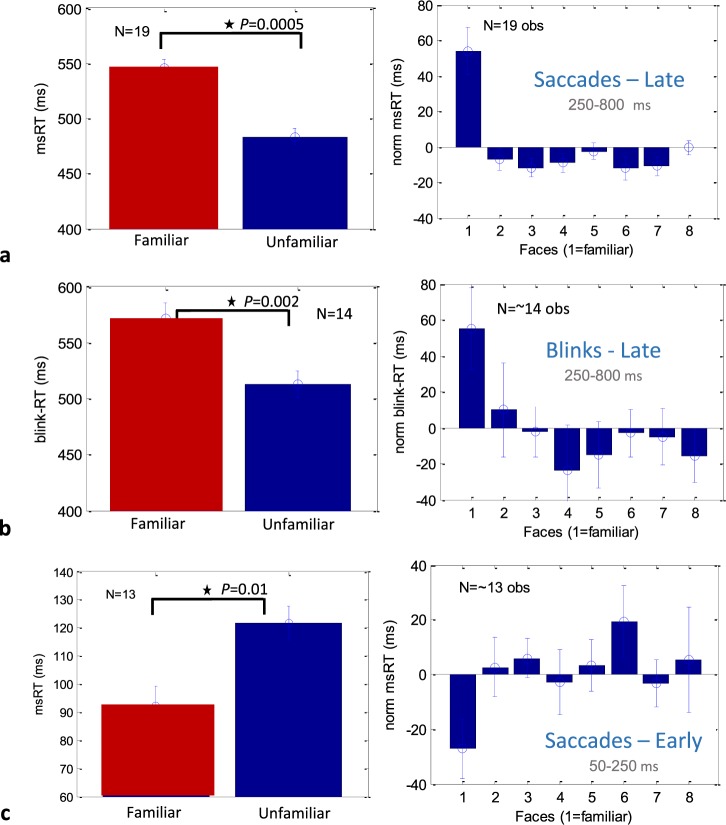
Group averages of microsaccade RT (msRT) for the familiar face compared to each of the non-familiar faces (right column, face 1 = familiar compared to faces 2–8) and their average (left column). The microsaccade and blink RTs were averaged and normalized (demeaned) per observers, then averaged across observers, with error bars denoting 1SE across observers (see Methods), with additional adjustment by adding the grand average (left column only). In (**a**,**b**), RT values were computed in the inhibition release period (250–800 ms), while the onset of inhibition (**c**) was computed in an early window (50–250 ms). The group average significance (nonparametric permutation test, see Methods) is indicated for each condition on the left column. Only N = 14 observers contributed data to the eyeblink RT averages due to the lack of eyeblinks in the specified time-period. Similarly, data from only N = 13 observers was available for estimating the onset of inhibition (see Results). Note the longer microsaccade (**a**) and blink (**b**) inhibition, and the earlier inhibition onset (**c**) for the familiar face.

We conducted a second analysis of the data using a discrete temporal measure, which we previously introduced for assessing contrast sensitivity and low-level processing^12,13^ and later applied it to language processing^8^. These measures, termed microsaccade and blink RT (response time, msRT, and bkRT, respectively), were computed by averaging the onset times of the first microsaccade or blink in a specified temporal window following stimulus onset: early (50–250 ms) around the onset of inhibition, and late (250–800) around its release, as shown in Fig. 2 (see Methods). These windows were roughly similar to those used in our previous studies^12,13^ and were derived from the rate modulation functions to capture the two phases of inhibition, onset and release. The results of this analysis are shown in Fig. 3. Figure 3a right panel compares the group’s average msRT at the late window, for the familiar (#1) and each of the unfamiliar faces (#2–8). The data were normalized per observer by subtracting the average msRT for all faces. As shown, the msRT for the familiar face was ~60 ms longer than for each of the other faces, with a highly significant group average effect of 73 ms when comparing the familiar face to the average of all non-familiar faces (Fig. 3a, left panel, p = 0.0005 in a non-parametric permutation test, see Methods). These results were not very specific to the choice of the window, and similar but more noisy results were obtained for a window starting at stimulus onset (see Fig. S3).

The results for the blink RT computed in the same late temporal window are shown in Fig. 3b. These results were very similar to those obtained with microsaccades, with a significant group average difference of 78 ms (p = 0.002, non-parametric permutation test, Fig. 3b left panel), and a difference between the familiar face and each of the non-familiar faces of about 60 ms (Fig. 3b, right panel). Only ~14 observers (on average, across the different faces) contributed to the group averages in this analysis due to zero blinks in the specified time window (see Methods).

Finally, the results for the early temporal window (50–250 ms) corresponding to the onset of inhibition are shown in Fig. 3c for microsaccades (blink rates approached zero in this window). In this early window, we averaged the onsets of the last microsaccade rather than the first, to capture the onset of inhibition (but only one microsaccade if any were typically found, see Methods). Owing to the sparse and limited microsaccade data in this early window, for some observers many faces could not be measured at all; therefore, we limited the analysis to observers with 3 or more faces with measured microsaccade RT, resulting in N = 13 observers. We found that the msRT (the average time of the last microsaccade in the early window), presumably related to the onset of inhibition, was ~30 ms faster for the familiar compared with the non-familiar faces (~90 ms and ~120 ms, respectively); this was true for both the group average (Fig. 3c left panel) and for the different face images (right panel). The group average effect was found to be significant using a non-parametric permutation test (p = 0.01, see Methods). Note that this early effect cannot be seen in the rate modulation functions (Fig. 2a), probably due to the floor effect introduced by subjects with fewer microsaccades, which were not included in the msRT calculation.

### The effect of familiarity for individual observers

**Figure 4:**
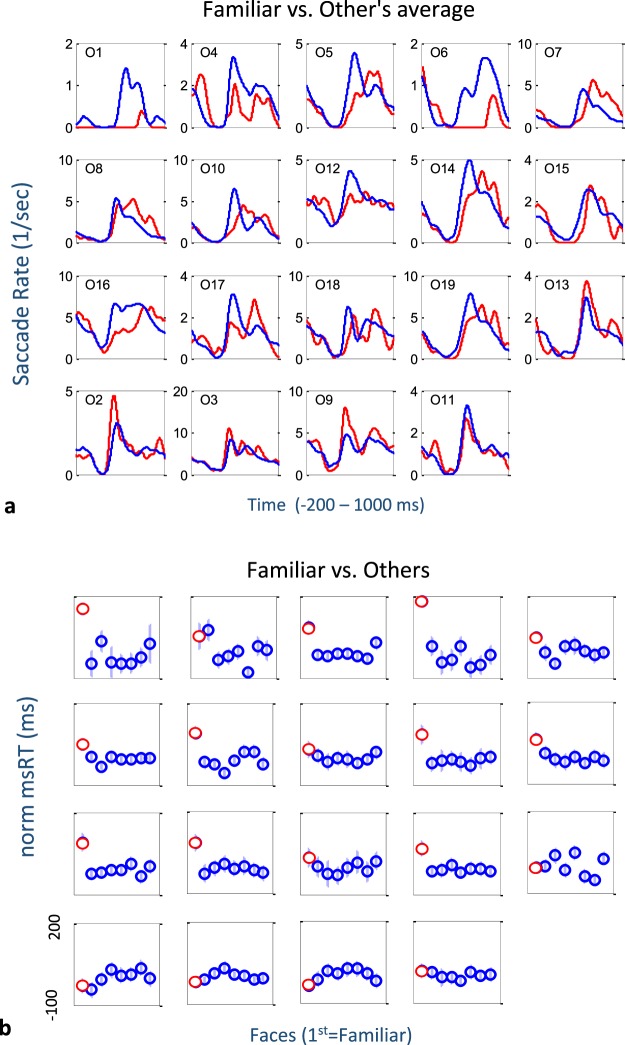
Individual observer data. (**a**) Microsaccade rate modulation functions for each observer, for the familiar face (red) compared to the average of all other faces. (**b**) Normalized msRT values in the release interval (250–800 ms post stimulus) for each observer and face (1–8, 1 = familiar in red). In both cases, data were ordered so that observers showing the group average effect appear in the first 3 rows. Note the delayed release from inhibition (**a**) and the typically longer msRT (**b**) for the familiar face compared to the other faces.

**Figure 5:**
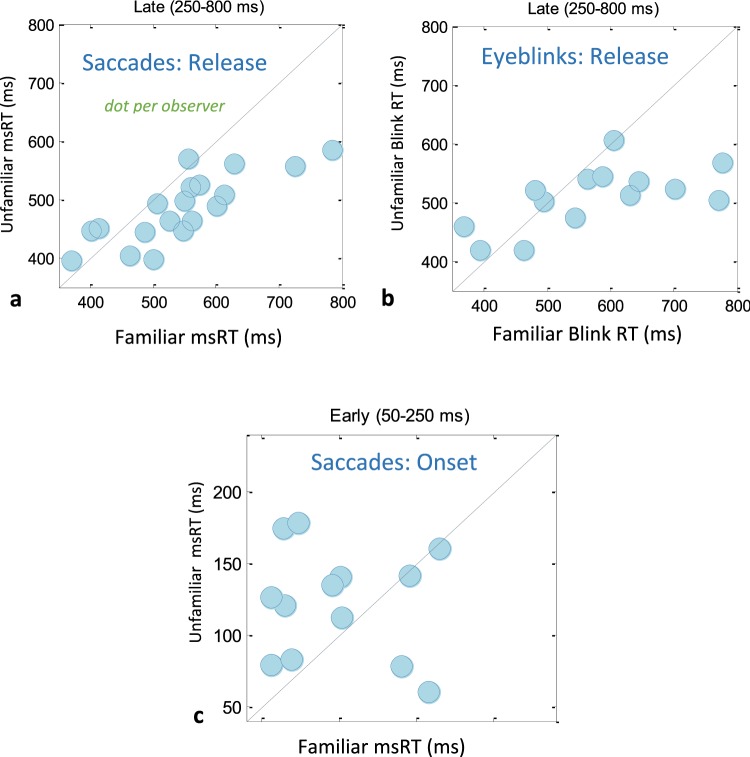
The relation between the oculomotor inhibition for familiar and unfamiliar faces. Observer scatter plots show the oculomotor RT for the familiar face on X and average of all unfamiliar faces on Y, with each dot representing one observer. The diagonal line represents the points of equality. (**a**) Microsaccade RT for the inhibition release (250–800), (**b**) Eyeblink RT for the inhibition release, (**c**) microsaccade RT for the onset of inhibition (50–250 ms). The results for eyeblinks do not include all observers because of a lack of blinking in the specific time-period investigated (see Methods). Similarly, the onset of microsaccade inhibition (**c**) includes only 13 observers. Note that in (**a**) and (**b**), the majority of the dots reside in the lower-right half of the figure, indicating prolonged inhibition for the familiar, while earlier inhibition onset for the familiar is indicated by the shorter msRT for the familiar (**c**).

We further analyzed the results for each observer separately (Figs 4 and 5). In Fig. 4a, we show the microsaccade rate modulation functions for each of the 19 observers and compared the rate time course for the familiar (red) and the average of the unfamiliar (blue). The graphs were scaled to the individual range of rates, and these rates varied across observers. We arranged the order of observers such that the first rows are more similar to the group average. As shown, in the first 3 rows (15 observers), the microsaccade inhibition release in response to a familiar face was delayed and “smeared”. The observers in the last row of Fig. 4a exhibited a different pattern, with a larger inhibition release response for a familiar face.

Figure 4b shows the normalized msRT values of the release from inhibition for each observer, like those shown in Fig. 3a’s right column for the group. The observers are in order, as in Fig. 4a, with the first 3 rows (excluding one) exhibiting a longer msRT for a familiar face compared with all other faces. We computed the p-value for the difference between the familiar and the average of the non-familiar faces for each of the observers using the non-parametric permutation test as done for the group (see Methods). We found that for 14 of the 19 observers the p-value was smaller than 0.05 (17 were below 0.15).

Figure 5 shows diagonal scatter plots of the oculomotor RT values (microsaccades, blinks) of the familiar face vs. the average of the unfamiliar faces, with one marker per observer. Figure 5a shows the msRT in the inhibition release period (250–800 ms). As shown, 15 of the 19 observers fell below the diagonal line, and the remaining 4 were above it, implying that a longer msRT for the familiar is a common property for most observers. Similar results were found for the blinks (Fig. 5b), although only 14 of the 19 observers had enough blinking for this analysis (see Methods, and Results above); however, 4 of them did not display this effect.

Finally, Fig. 5 shows the microsaccade RT for the early time interval (50–250 ms), which is assumed to quantify the onset of inhibition (see Methods). Since in many trials no microsaccade was found, we included only observers that had measures for more than 2 unfamiliar images. This resulted in only 13 observers. As shown, 9 of the observers were above the diagonal line, implying that their onset of inhibition was earlier, about 90 ms, on average.

### The effect of familiarity on the OMI change over time

**Figure 6:**
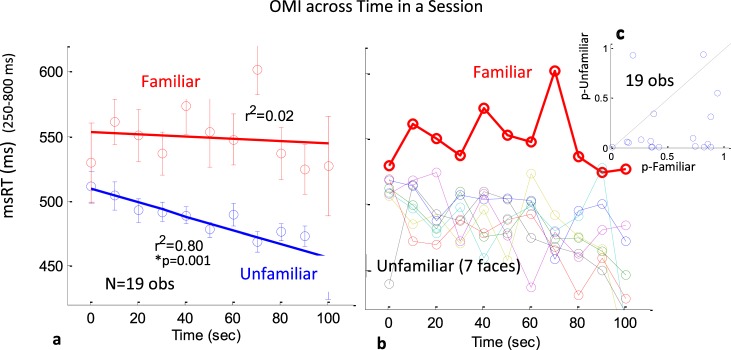
The OMI across time in a session for the familiar and unfamiliar. The msRT for the release period (250–800 ms) is computed in 10 s bins across the experimental runs of ~100 s each, and averaged across observers. Error bars are 1SE after normalization by demeaning. (**a**) Familiar face compared to the average of all unfamiliar faces; (**b**) unfamiliar faces plotted sepearately; (**c**) standard p-vlaues for the correlations computed for each observer for the famliar and the average of the unfamiliar, and shown in a scatter plot. As shown, the unfamiliar msRT was reduced steadily across the ~100 s runs (R = ~−0.9, p = 0.001 in a permutation test) while this was not the case for the familiar face. The plot in (**b**) shows that this was true for the individual unfamiliar faces, and (**c**) shows that this was true for 15 of the 19 observers. This demonstrates another difference in the processing of the familiar and unfamiliar faces, which calls for further investigation.

A change of the OMI over time within a session could be indicative of some adaptation that typically occurs with repeated presentation of the same stimulus, or alternatively, the buildup of anticipation of the “discovered” familiar face. To investigate this, we computed the msRT as a function of time within the ~100 sec experimental runs in 10 sec bins. We averaged the data across the 3 runs since there was no significant difference between them. The results appear in Fig. 6. As shown in (a) and (b), the msRT decreased by ~50 ms over the session period, but only for the unfamiliar (r^2^ = 0.8, *p* = 0.001, via permutation test). In contrast, the familiar face remained with longer OMI and did not change systematically. This was not because the unfamiliar were many – the same decrease was found for the individual unfamiliar faces (b). This was also a property common to most observers (14 of 19) as shown on (c).

## Discussion

We investigated the effect of face familiarity on microsaccade and blink inhibition by presenting barely visible faces at a regular 1 Hz repetition rate and with passive viewing. Our results revealed that the microsaccade inhibition time course was modulated by familiarity, where briefly flashed and masked familiar faces evoked earlier and longer microsaccade inhibition compared with unfamiliar faces (Figs 2a and 3). These findings indicate, for the first time, that microsaccades are sensitive to familiarity.

In addition to the longer microsaccade inhibition, familiarity also induced a delay in the timing of spontaneous eye blinks. Similar to our previous study^12^, the regular 1 Hz stimuli caused entrainment of the eye blinks (Fig. 2b), with almost totally inhibited blinking prior to and immediately after the stimulus onset, and a high blink probability around 400 ms post-stimulus. This release from inhibition was significantly delayed for the familiar compared with the unfamiliar faces; this indicates the sensitivity of spontaneous eye blink timing to familiarity.

Taken together, these results provide further support to the idea of a common oculomotor inhibition mechanism (OMI) that presumably turns off oculomotor events while processing previous stimuli^12^, processing that is affected by familiarity.

### Familiarity and perceptual saliency: an oculomotor marker

Saliency appears to be a major factor that determines the properties of OMI; however, there are different types of saliency, i.e., things that attract attention, and which affect the OMI differently. Whereas sensory saliency such as higher contrast results in earlier and shorter OMI^13^, oddballs or surprising stimuli tend to prolong it^7^. We found that familiar stimuli (faces) behave as perceptual oddballs and result in prolonged OMI. It appears that longer OMI is associated with longer processing times, since paradigms that involve stimulus categorization prolong the inhibition^8,23^. For example, in a recent study of language processing in which observers were required to categorize words and non-words^8^, non-words containing real word roots, produced longer OMI than did other non-words containing invented roots, presumably due to the longer processing time for discrimination.

Additional insight is provided by examining the change of the msRT over time within a session (Fig. 6). We found that it was shortened by 50 ms over ~100 sec experimental runs for the unfamiliar but not the familiar faces. In a previous study^39^, we found that a repeated presentation of the same stimulus resulted in shortening of the OMI (expressed by the msRT for its release), while changes (oddballs) caused prolonging of the OMI. This suggests that the reduction in the msRT over time for the unfamiliar reflects a perception of all these faces as one repeating stimulus, and the familiar as an oddball. Next, we try to account for the main findings by suggesting a working memory encoding process that determines the OMI.

### Familiarity and OMI: a working memory encoding hypothesis

We propose here that for a large set of stimuli, and specifically for the facial stimuli used in the current study, the primary processing stage that determines the OMI is working memory (WM) encoding, which varies in time and is affected by familiarity. Working memory is a cognitive system used for temporarily holding information available for processing^40,41^; the encoding phase in working memory is a critical component of it^42,43^. WM-load was found to reduce microsaccade rate^44^. Our hypothesis refers here to the encoding phase of transient events. First, this hypothesis is consistent with the evidence for shorter OMI with bottom-up saliency (e.g., contrast) because WM encoding should be faster for more coherent (or strongly activated) representations. Second, a longer encoding time is expected for novel events involving an update in stimulus properties, compared to repeating the same stimulus (which requires no WM-update), accounting for the simple oddball and serial dependence effects. Third, a longer encoding time is expected for more important/significant stimuli because of the deeper and richer associations made between the stimulus and other knowledge representations^41^. Since face recognition is based on spontaneous activation of a wide range of personal knowledge and emotions^24,26^, we propose that the longer OMI we observed for familiar compared with unfamiliar faces is due to a richer and longer WM encoding for the familiar faces.

But why don’t we get a prolonged OMI for the unfamiliar faces due to their novelty? This could be due to the masking we used, which reduced visibility or awareness. It has been suggested that a cortical recurrent-loop mechanism which crosses a threshold and “ignites”, leads to content-specific awareness^45^, and presumably to WM encoding. In our case, the unfamiliar faces may have failed to “ignite” and their WM encoding could have been limited. Our finding that the repeated presentation of the unfamiliar faces resulted in shortening of the OMI (Fig. 6) support this interpretation by suggesting that the unfamiliar faces have been treated as a repeated presentation of the same stimulus, rather than as novel faces. On the other hand, we verified that the familiar face had been encoded to WM, by a verbal report of all the observers (see Results). Finally, a recent ERP study with RSVP of faces that, like our study, limits visibility by masking, found that famous but not unfamiliar faces could break through this masking into awareness and produce measurable ERP^20^, presumably because the novel (unfamiliar) faces were not perceived sufficiently to be encoded into WM.

### Early and late processing of familiarity

Whether familiarity detection is an early or late process has been studied extensively, mainly for faces. Our results were divided into an early and late analysis windows, corresponding to the onset and release of inhibition, as done in our previous studies^8,12,13^. We found that for microsaccades the inhibition onset was earlier for the familiar, as reflected by the shorter average latency of the last microsaccade in the window of 50–250 post stimulus (Figs 3c and 4c), ~90 ms and ~120 ms for the familiar and unfamiliar, respectively. This early effect, though significant, is not as strong and convincing as the late effect (larger p-value, see Results, Fig. 3a vs. 3c) because of a reduced sample size (n = 13) due to the lack of microsaccades in the early time period for some participants, and the difficulty in deriving accurate time measures from the lack of events (microsaccades) compared to occurring events.

Nevertheless, we believe that this result is indicative of the fast and early recognition of familiarity, similar to the microsaccade inhibition effect obtained previously for auditory pattern categorization^23^. The current evidence on brain responses to face familiarity suggests mainly a late ERP response, including the N250 component and the more general attention-related P300 response^46–49^. These late responses, and primarily the P300 component, appear to be related to our late familiarity effects (250–800 ms, Figs 3a,b and 5a,b), as previously suggested for oddballs^7^.

However, additional recent studies found an earlier effect of face familiarity, including the modulation of the N170 response when a familiar face was expected^50^, a rapid choice saccade to a familiar face in 180 ms^49^, and accurate familiar face detection at a rapid 7 faces/s presentation rate^51^ (although this could also reflect a processing pipeline). In our experiment, expectation could have played a role because we used repeated presentations of one familiar face among unfamiliar faces, and once the familiar face was discovered (e.g., in a late processing stage), the viewer might have developed an expectation that primed early selection. We did not find a change over time in the OMI for the familiar face to provide evidence for the buildup of this expectation (Fig. 6 and results), but this could still be the case if the buildup was quick. In general, the early recognition of the familiar face is consistent with models of early auditory selection, which account, for example, for the cocktail party effect^52^, implicating early mechanisms of MGM and inferior colliculus, which might similarly apply to vision.

Given the early and late effects we measured, it is possible to sketch a two-stage process involved in detecting face familiarity, and possibly other types of familiarity. The first stage, which corresponds to the inhibition onset we found, is early in time (around 100 ms) and is responsible for rapid detection of stimuli of potential valence. This could be implemented by a fast and crude readout mechanism, like that suggested for the first stage in object recognition^53,54^. A second processing stage comes later, possibly using feedback, to fully identify the familiar stimulus; its end is denoted by the release from oculomotor inhibition. Such a scheme for processing face familiarity is supported by a recent ERP study demonstrating an early and coarse fast stage starting around 140 ms and a more refined but slower stage occurring after 200 ms^55^.

### Application to non-facial stimuli and concealed information

It is not yet known whether the OMI markers of face familiarity also apply to non-facial stimuli, such as familiar items or text words; however, this seems very likely. First, we obtained preliminary results of prolonged OMI with familiar text names. Second, several studies of concealed information detection demonstrate ERP response specificity to familiar text, including a P3 response for barely visible personal names in RSVP^18,19^ and a P300 response for familiar text words^32,56^.

In the current study, we used barely visible stimuli, similar to Bowman *et al*.^19,18^ in order to increase the difference between the familiar and unfamiliar faces, since previous studies suggest that only familiar faces are processed unconsciously or on the verge of awareness^24,26,27,57,58^ (see also Introduction). It is not yet clear whether the OMI can react selectively to subconscious (totally invisible) stimuli, and more specifically, to the familiarity of invisible faces. Under some conditions, the oculomotor system may react without awareness^58^, although for contrast, the OMI was found to co-vary with conscious detection^37^.

The use of barely visible stimuli has another potential advantage in detecting concealed information. The facial memory that was uncovered in our test was revealed in an automatic and involuntary manner, tapping into an internal cognitive process in the absence of the subject’s attention or conscious control. This may minimize irrelevant biases and has potential implications in legal procedures, for minimizing misidentifications and preventing false convictions.

## Methods

### Participants

Nineteen observers (ages 30–45 and one aged 54) with normal or corrected-to-normal vision participated in the experiments. They were recruited among university students and friends and all were familiar or became familiar with the experimenter (GR). The study including the experimental protocol was approved by the ethics committee (IRB) of the Haifa University, and the methods were carried out in accordance with the IRB guidelines and regulations. Informed consent was obtained from all participants.

### Apparatus

Stimuli were displayed on a 22-inch CRT monitor, running at a 100-Hz refresh rate with 1024 × 768 pixel resolution occupying a 33.4° × 25.4° area. The background luminance was 3.2 cd/m^2^. The experiments were administered in dim light. Eye movements were recorded monocularly with an Eyelink 1000 infrared system (SR Research, Ontario, Canada) with a sampling rate of 500 Hz. Head movements were limited by a chin and forehead rest, placed 60 cm from the screen. Recording was performed from the right eye, although viewing was binocular. A standard 9-point calibration was performed before each session. Stimuli were presented using an in-house-developed platform for psychophysics and eye-tracking experiments (PSY) developed by Y.S.B., running on a Windows PC.

### Stimuli and procedures

Both familiar and unfamiliar faces were presented in an involuntary familiarity detection paradigm of one in eight (one familiar and 7 unfamiliar), like in an identification lineup. Participants passively viewed sequences of stimuli with no instructions other than fixating at a central static fixation point (0.128 in diameter) and paying attention to the presented stimuli. The stimuli consisted of 96 presentations or epochs per run shown at 1 Hz, 3 runs per participant. An example of a stimulus sequence presented in one epoch is shown in Fig. 1. It starts with a 830 ms fixation, a monochromatic facial image (one of 8) flashed for 10 ms, a blank screen (60 ms), and two successive colorful “relaxing” images, 50 ms each, selected at random from a set of 30 images; all images were 360 × 480 pixels in size. Each face (among the 8 faces) was presented 12 times in random permutation order, with a total of 96 per run. Six of the observers also performed an additional control experiment, which was identical to the main experiment, except for the absence of the masks.

The participants were first tested on a universally familiar Hollywood star, Daniel Craig (“James Bond”), and asked about their familiarity with him only after the experiment. If they reported “unfamiliar”, they were asked about their familiarity with the American TV series Seinfeld. If they reported “familiar”, they were tested on “Kramer”, one of the actors in this TV series. If they replied “unfamiliar”, they were tested on the picture of the experimenter, GR. In total, 12 of the 19 participants were tested on a universally familiar face (3 on “Kramer”), and 7 on an individually familiar face (the experimenter, GR). These two types of familiarity did not produce significant differences in the results (see Results).

An additional complementary experiment was conducted at a later stage with a new group of 10 observers who watched an identical stimulus sequence, except that 3 mask SOA values were used, 30 ms (fast), 70 ms (as in the main experiment) and 100 ms (slow). The participants were required to rate visibility for each stimulus presentation via 3 keys, 1 for “invisible”, 2 for “barely visible” and 3 for “visible”. Each observer was tested in two runs of 72 presentations each. At the end of the experiment, participants were asked to report what they observed.

### Data analysis

We compared event-related measures of microsaccades and eye blinks in response to a *familiar face* vs. *unfamiliar faces*. They include rate modulation functions and oculomotor RT measures (microsaccades, eye blinks), as used in our previous studies^8,12,13^ and are described next. Data analysis was carried out using in-house software written in Matlab (The Mathworks, Natick, MA), developed by Y.S.B.

#### Microsaccade and blink detection

Microsaccades were detected using the algorithm introduced by^15^ as implemented in^8,13^. Details are repeated here for completeness. Raw data were first smoothed using local linear regression fitting (LOWESS method, span of 25 ms) to optimize microsaccade extraction. Microsaccades were detected as intervals in which the velocity exceeded a threshold defined as eight median standard deviations of the horizontal and vertical velocities (λ = 8). The minimal microsaccade duration was set at 9 ms. The permitted velocity range was 8°/s–150°/s and the permitted amplitude range was 0.08°–2°. Eye movements outside these ranges were rejected. The rejection rate varied across participants and was in the range of 0–33%, with an average of 4.1%. When microsaccades were analyzed, periods of missing data, such as during blinks, were locally discarded from further analysis with an additional margin of 100 ms, without discarding the whole epoch.

Eye blinks were detected as in^8^. Blink periods were first defined as zero pupil size, producing approximate events of eyes closed (transition to zero) and eyes open (changed from zero). Since a blink is typically preceded by a vertical eye movement, the vertical trace was further analyzed in a local window of 100 ms prior to an approximate onset and 150 ms after an approximate offset. The first third of the local window was used to calculate a baseline for transient changes in the vertical trace. The new blink onset was then defined as the time when the change in the vertical trace passed a threshold of 4 standard deviations of that baseline. Blink offset was defined similarly. Lastly, blinks shorter than 250 ms or longer than 700 ms were rejected as possibly reflecting measurement noise. Following extraction, the recorded data were divided into epochs time-locked to stimulus onset, such that each epoch represented one experimental trial.

#### Microsaccade and blink rate modulation

The rate modulation function for both microsaccades and blinks was calculated as in^8^. Rates were computed by convolving a raw rate estimate of one microsaccade (or blink) per sample duration at the time of onset with a causal kernel^23^. The rates were first averaged across epochs within participants, and then across participants, to compute the event-related modulation of microsaccades (or blinks) with equal contribution from each participant.

#### Microsaccade and blink reaction time (RT)

Quantitative measures for the microsaccade- (or blink-) inhibition duration were computed using a method introduced in^12,13^. Microsaccade RT (msRT) was calculated per epoch as the latency of the first microsaccade after stimulus onset in two specified time windows: 50–250 ms as the inhibition onset interval, and 250–800 ms as the inhibition release interval. In the early interval, the last (rather than the first) microsaccade was selected, although this change had a negligible effect since microsaccades in the early interval were rare. The Blink RT (bkRT) was similarly calculated in the same time intervals. Epochs with no microsaccades or blinks in the specified window were not included in this calculation. In computing error bars for the RT values averaged across subjects, we applied the Cousineau method, which controls the between-subject variance and allows a better representation of within-subject effects^59^. In this method, data are first normalized by subtracting each subject’s mean RT and adding the group mean RT across all conditions and subjects. The standard error is calculated over the normalized data, and is multiplied by Morey’s correction factor^60^ (√(n/(n − 1)), which equals a negligible √(19/18)) for n = 19 subjects. In some cases, less than the full number of subjects was averaged due to the lack of microsaccades or blinks in the specified window for any of the relevant epochs. In all cases, however, the actual number of subjects is indicated in the figures.

### Statistical assessment

We used nonparametric permutation tests^61^ to test the difference in msRT between the familiar and unfamiliar faces. For each test, we randomly permuted the labels (1–8) of the epochs (1,000 permutations) and recalculated the group average msRT. We then computed the p value as the fraction of permutations in which the original effect size (i.e., the difference between the conditions divided by the pooled standard deviation) was exceeded by the effect size of the permuted data. We used the same procedure to assess the significance of the bkRT effect. For assessing the significance of the correlation between time bin and msRT (Fig. 6), we used a similar permutation test, except that the time bin was permuted and the correlation was used as the effect size, i.e. we computed the number of random permutations for which the correlation equal or higher than the original.

For assessing the significance in the difference in the microsaccade rate modulation functions of familiar vs. the average of the unfamiliar faces, we used a nonparametric cluster-based randomization test^8,23,62^ as follows: For each time point, we calculated a paired t-test between the two rate functions. We then identified clusters of adjacent time points showing a significant t-value, and calculated cluster-level statistics by summing all the t-values within a cluster. Then we randomly permuted (1,000 permutations) the labels of the data (i.e., whether each value belonged to the familiar vs. unfamiliar faces average), recalculated the cluster-level t-value, and generated a histogram of the test statistics across the permutations. We then computed the p value as the fraction of permutations in which the original cluster-level t-value was exceeded by that of the permuted data.

## Supplementary information

Supplementary Figures

## Data Availability

The stimuli are public and could also be obtained upon request. The data are presented graphically in the manuscript in detail, including all the individual results, with numerical representations of these graphs obtained upon request as applicable.

## References

[1] Barlow, H. B. Eye movements during fixation. *The Journal of Physiology*, **116**, 290–306 (1952) 10.1113/jphysiol.1952.sp004706 PMC1392140 14939180

[2] Steinman RM, Haddad GM, Skavenski AA, Wyman D (1973). Miniature eye movement. Science.

[3] Hafed ZM, Clark JJ (2002). Microsaccades as an overt measure of covert attention shifts. Vision Res..

[4] Yuval-Greenberg S, Merriam EP, Heeger DJ (2014). Spontaneous Microsaccades Reflect Shifts in Covert Attention. J. Neurosci..

[5] Betta E, Turatto M (2006). Are you ready? I can tell by looking at your microsaccades. Neuroreport.

[6] Valsecchi M, Betta E, Turatto M (2007). Visual oddballs induce prolonged microsaccadic inhibition. Exp. Brain Res..

[7] Valsecchi M, Turatto M (2009). Microsaccadic responses in a bimodal oddball task. Psychol. Res..

[8] Yablonski, M., Polat, U., Bonneh, Y. S. & Ben-Shachar, M. Microsaccades are sensitive to word structure: A novel approach to study language processing. *Sci*. *Rep*. **7** (2017). 10.1038/s41598-017-04391-4 PMC5479819 28638094

[9] Bonneh Y, Adini Y, Fried M, Arieli A (2011). An oculomotor trace of cognitive engagement. J. Vis..

[10] Bonneh YS (2010). Motion-induced blindness and microsaccades: cause and effect. J. Vis..

[11] Rolfs M (2009). Microsaccades: Small steps on a long way. Vision Res..

[12] Bonneh, Y. S., Adini, Y. & Polat, U. Contrast sensitivity revealed by spontaneous eyeblinks: Evidence for a common mechanism of oculomotor inhibition. *J*. *Vis*. **16** (2016). 10.1167/16.7.1 27135194

[13] Bonneh, Y. S., Adini, Y. & Polat, U. Contrast sensitivity revealed by microsaccades. *J*. *Vis*. **15** (2015). 10.1167/15.9.11 26223023

[14] Rolfs M, Kliegl R, Engbert R (2008). Toward a model of microsaccade generation: The case of microsaccade inhibition. J. Vis..

[15] Engbert R, Kliegl R (2003). Microsaccades uncover the orientation of covert attention. Vision Res..

[16] Pastukhov A, Braun J (2010). Rare but precious: Microsaccades are highly informative about attentional allocation. Vision Res..

[17] Valsecchi M, Turatto M (2007). Microsaccadic response to visual events that are invisible to the superior colliculus. Behav. Neurosci..

[18] Bowman, H. *et al*. Subliminal Salience Search Illustrated: EEG Identity and Deception Detection on the Fringe of Awareness. *PLoS One* **8** (2013). 10.1371/journal.pone.0054258 PMC3553137 23372697

[19] Bowman, H., Filetti, M., Alsufyani, A., Janssen, D. & Su, L. Countering countermeasures: Detecting identity lies by detecting conscious breakthrough. *PLoS One* **9** (2014). 10.1371/journal.pone.0090595 PMC3946631 24608749

[20] Alsufyani A (2019). Breakthrough percepts of famous faces. Psychophysiology.

[21] Oleggio Castello VD (2017). M. I. The neural representation of personally familiar and unfamiliar faces in the distributed system for face perception. Sci. Rep..

[22] Di Oleggio Castello, M. V., Taylor, M., Cavanagh, P. & Gobbini, M. I. Idiosyncratic, Retinotopic Bias in Face Identification Modulated by Familiarity. **5** (2018). 10.1523/ENEURO.0054-18.2018 PMC6171739 30294669

[23] Widmann A, Engbert R, Schroger E (2014). Microsaccadic Responses Indicate Fast Categorization of Sounds: A Novel Approach to Study Auditory Cognition. J. Neurosci..

[24] Gobbini MI, Haxby JV (2007). Neural systems for recognition of familiar faces. Neuropsychologia.

[25] Schwedes C, Wentura D (2012). The revealing glance: Eye gaze behavior to concealed information. Mem. Cogn..

[26] Axelrod V, Bar M, Rees G (2015). Exploring the unconscious using faces. Trends Cogn. Sci..

[27] Gobbini MI (2013). Prioritized Detection of Personally Familiar Faces. PLoS One.

[28] Henson RN, Mouchlianitis E, Matthews WJ, Kouider S (2008). Electrophysiological correlates of masked face priming. Neuroimage.

[29] Hannula DE, Baym CL, Warren DE, Cohen NJ (2012). The eyes know: Eye movements as a veridical index of memory. Psychol. Sci..

[30] Hannula DE, Ryan JD, Tranel D, Cohen NJ (2007). Rapid onset relational memory effects are evident in eye movement behavior, but not in hippocampal amnesia. J. Cogn. Neurosci..

[31] Lancry-Dayan, O. C., Nahari, T., Ben-Shakhar, G. & Pertzov, Y. Do You Know Him? Gaze Dynamics Toward Familiar Faces on a Concealed InformationTest. *J*. *Appl*. *Res*. *Mem*. *Cogn*. 1–12, 10.1016/j.jarmac.2018.01.011 (2018).

[32] Meixner JB, Rosenfeld JP (2011). A mock terrorism application of the P300-based concealed information test. Psychophysiology.

[33] Rosenfeld JP, Hu X, Pederson K (2012). Deception awareness improves P300-based deception detection in concealed information tests. Int. J. Psychophysiol..

[34] Linden DEJ (2005). The P300: Where in the brain is it produced and what does it tell us?. Neuroscientist.

[35] Rosenfeld, J. P. P300 in detecting concealed information. *Mem*. *Detect*. *Theory Appl*. *Concealed Inf*. *Test* 63–89 (2011).

[36] Valsecchi M, Dimigen O, Kliegl R, Sommer W, Turatto M (2009). Microsaccadic inhibition and P300 enhancement in a visual oddball task. Psychophysiology.

[37] White AL, Rolfs M (2016). Oculomotor inhibition covaries with conscious detection. J. Neurophysiol..

[38] Kliegl R, Rolfs M, Laubrock J, Engbert R (2009). Microsaccadic modulation of response times in spatial attention tasks. Psychol. Res..

[39] Bonneh Y (2013). Microsaccade latency uncovers stimulus predictability: Faster and longer inhibition for unpredicted stimuli. J. Vis..

[40] Baddeley, A. Working memory. *Science (80-*.*)*, 10.1126/science.1736359 (1992).

[41] Davelaar, E. J., Goshen-Gottstein, Y., Ashkenazi, A., Haarmann, H. J. & Usher, M. The demise of short-term memory revisited: Empirical and computational investigations of recency effects. *Psychol*. *Rev*., 10.1037/0033-295X.112.1.3 (2005). 10.1037/0033-295X.112.1.3 15631586

[42] Brodski-Guerniero A (2017). Information theoretic evidence for predictive coding in the face processing system. J. Neurosci..

[43] Manuscript, A. The Neural Correlates of Visual WM. **49**, 1527–1536 (2012).

[44] Dalmaso M, Castelli L, Scatturin P, Galfano G (2017). Working memory load modulates microsaccadic rate. J. Vis..

[45] Moutard C, Dehaene S, Malach R (2015). Spontaneous Fluctuations and Non-linear Ignitions: Two Dynamic Faces of Cortical Recurrent Loops. Neuron.

[46] Eimer M (2000). Event-related brain potentials distinguish processing stages involved in face perception and recognition. Clin. Neurophysiol..

[47] Andrews S, Burton AM, Schweinberger SR, Wiese H (2017). Event-related potentials reveal the development of stable face representations from natural variability. Q. J. Exp. Psychol..

[48] Huang W (2017). Revisiting the earliest electrophysiological correlate of familiar face recognition. Int. J. Psychophysiol..

[49] Di Oleggio Castello MV, Gobbini MI (2015). Familiar face detection in 180ms. PLoS One.

[50] Johnston P, Overell A, Kaufman J, Robinson J, Young AW (2016). Expectations about person identity modulate the face-sensitive N170. Cortex.

[51] Hacker, C. M., Meschke, E. X. & Biederman, I. A face in a (temporal) crowd. *Vision Res*., 10.1016/j.visres.2018.02.007 (2018). 10.1016/j.visres.2018.02.007 29555301

[52] McLachlan N, Wilson S (2010). The Central Role of Recognition in Auditory Perception: A Neurobiological Model. Psychol. Rev..

[53] Bar M (2004). Visual objects in context. Nat. Rev. Neurosci..

[54] Bar, M. The Proactive Brain. *Predict*. *Brain Using Our Past to Gener*. *a Futur*. 1235–1243, 10.1093/acprof:oso/9780195395518.003.0010 (2011).

[55] Barragan-Jason G, Cauchoix M, Barbeau EJ (2015). The neural speed of familiar face recognition. Neuropsychologia.

[56] Labkovsky E, Rosenfeld JP (2012). The P300-based, complex trial protocol for concealed information detection resists any number of sequential countermeasures against up to five irrelevant stimuli. Appl. Psychophysiol. Biofeedback.

[57] Wood SMW, Wood JN (2015). Face recognition in newly hatched chicks at the onset of vision. J. Exp. Psychol. Anim. Learn. Cogn..

[58] Spering M, Carrasco M (2015). Acting without seeing: Eye movements reveal visual processing without awareness. Trends Neurosci..

[59] Cousineau D (2005). Confidence intervals in within-subject designs: A simpler solution to Loftus and Masson’s method. Tutor. Quant. Methods Psychol..

[60] Morey RD (2008). Confidence Intervals from Normalized Data: A correction to Cousineau (2005). Tutor. Quant. Methods Psychol..

[61] Efron B, Tibshirani RJ (1993). An Introduction To The Bootstrap. Journal of the American Statistical Association.

[62] Maris E, Oostenveld R (2007). Nonparametric statistical testing of EEG- and MEG-data. J. Neurosci. Methods.

