# Supplementary material for: Familiarity revealed by involuntary eye movements on the fringe of awareness
Source: Sci Rep. 2019 Feb 28;9:3029. doi: 10.1038/s41598-019-39889-6 (PMC6395845; doi:10.1038/s41598-019-39889-6)
Supplement: Supplementary file 1 — Supplementary Figures [file 41598_2019_39889_MOESM1_ESM.docx]

**Familiarity revealed by involuntary eye movements on the fringe of awareness**

Gal Rosenzweig and Yoram S. Bonneh

**Supplementary figure S1:** The effect of masking on the OMI changes with familiarity, preliminary data from 6 observers.

**Supplementary figure S2:** The effect of SOA on the level of stimulus visibility.

**Supplementary figure S3:** The sensitivity of the msRT measure to the choice of the temporal window.

6 observers in different colors

Not Masked

Masked

Familiar

Familiar

**b**

**a**

**Figure S1**. The effect of masking on the OMI changes with familiarity, preliminary data from 6 observers. The figure shows the normalized msRT for the release period (in the range 250-850 ms) as in Figure 3a, for the standard masking paradigm (see Methods) in (a) and an identical experiment (same observers) without the maskers in (b) for each of the observers superimposed. As shown, the unmasked results are more variable or noisy, with several faces (in addition to the familiar) inducing above-average msRT. This demonstrates the potential benefit of masking.

1=Invisible 2=Barely 3=Visible

**Figure S2.** The effect of SOA on the level of stimulus visibility. Results from a complementary experiment for verifying the visibility level in the main paradigm. Observers (n=10) rated visibility by pressing buttons (1=Invisible, 2=Barely visible, 3=Visible), and the average rating thus represents the level of visibility, plotted for the different levels of SOA. Note that the stimuli were "barely visible" on average.

Familiar

Familiar

**b**

**a**

**Figure S3.** The sensitivity of the msRT measure to the choice of the temporal window.
(**a**) The group results (n=19) for the optimal window for OMI release (250-800ms) as in Figure 3a, are compared with a non-optimal window of 0-800 ms (**b**). As shown, the effect size of the longer msRT for the familiar is smaller for the non-optimal time window, presumably because of including saccades that occured before the inhibition fully kicked in. In comparing the familiar with the average of the non-familiar we get a difference of 60ms for the release period (250-800ms), equivalent to 8 SE, and 40 ms for the full period of 0-800, equivalent to 4.5 SE (extending the range to 0-1000ms makes the difference even smaller). Similarly, the effect for the eye blinks changed from 60ms (~= 4.7 SE) to 47ms (~= 2.8 SE).
